# MiR-92a modulates proliferation, apoptosis, migration, and invasion of osteosarcoma cell lines by targeting Dickkopf-related protein 3

**DOI:** 10.1042/BSR20190410

**Published:** 2019-04-26

**Authors:** Haiyang Yu, Hang Song, Li Liu, Shuo Hu, Yuxin Liao, Gang Li, Xiao Xiao, Xin Chen, Shisheng He

**Affiliations:** 1 Orthopedic Department, Shanghai Tenth People’s Hospital, Tongji University School of Medicine, 301 Yanchang Road, Shanghai 200072, China; 2 Spinal Pain Research Institute, Tongji University School of Medicine, 301 Yanchang Road, Shanghai 200072, China; 3 Department of Laboratory Medicine, Shanghai Children’s Medical Center, School of Medicine, Shanghai Jiaotong University, Shanghai, China; 4 Department of Clinical Laboratory, Shanghai Fourth People’s Hospital, Tongji University School of Medicine, 1878 North Sichuan Road, Shanghai, 200081, China

**Keywords:** Apoptosis, DKK3, MiR-92a, osteosarcoma, Proliferation

## Abstract

Osteosarcoma (OS) is recognized as a common malignant tumor with a high trend of metastasis and diffusion. Despite the progresses that have been made in surgery, chemotherapy, and radiotherapy in the recent decades, the prognosis of patients with OS still remains poor. MiRNAs are being increasingly considered as new therapeutic targets for OS treatment. Our research aims to investigate the regulatory impact of miR-92a in the development of OS. Quantitative real-time PCR (qRT-PCR) results revealed that the expression of miR-92a was aberrantly overexpressed in human OS cell lines. By using cell counting kit-8 (CCK-8) assays, colony formation assays, flow cytometric analyses and Transwell assays, our data suggested that up-regulation of miR-92a promoted the proliferation, migration, and invasion of MNNG and U2OS cells, while inhibiting their apoptosis. In contrast, the knockdown of miR-92a effectively reversed these cellular biological behaviors. Furthermore, bioinformatics analysis indicated that Dickkopf-related protein 3 (DKK3) was a possible target of miR-92a. Subsequently, negative regulation of miR-92a on DKK3 was observed, which further supported the direct binding between them. In addition, silencing DKK3 rescued the inhibitory effect of miR-92a inhibitor on the development of OS. To sum up, our study revealed that miR-92a played a carcinogenic role in the growth of OS by promoting the tumorigenesis of OS cells via targeting of DKK3, thus revealing a new therapeutic target for OS.

## Introduction

 Osteosarcoma (OS) is the most common primary bone malignant tumor, predominantly occurring in children and adolescents [3]. It is characterized by high malignancy and metastatic potency, accounting for nearly 50% of metastatic bone sarcomas [11,19]. A combination of surgical resection, chemotherapy, and radiotherapy is the main treatment for OS, and has led to considerable progress in the development of therapeutic strategies for OS [31,25]. However, even with combined treatment, the 5-year survival rate of OS is still not optimistic [16]. Distant metastasis and local recurrence can still occur in a considerable number of patients, whose prognosis is extremely poor. Therefore, we urgently need to clarify the molecular mechanism of the occurrence, development, and metastasis of OS.

MiRNAs are a class of small non-coding RNAs that induce mRNA degradation or suppress mRNA translation by binding to the 3′-UTRs of target genes; thereby, participating in a variety of biological processes including tumorigenesis [14,1]. Accumulating evidence indicates that dysfunctional and dysregulated miRNAs play an important role in various human diseases, including most cancers, because they affect the translation and stability of oncogenes and tumor suppressors by targeting – and ultimately influencing – the biological capacity of cells [2,18,22]. In terms of OS, various miRNAs exhibit differential expression between tumor and normal tissues, including miR-144, miR-30a, miR-99b, miR-92a, miR-132, miR-422a, and miR-193a-5p [10,7,32]. Notably, miR-92a has proven to be a diagnostic tool for OS and other tumors [7,28]. Numerous investigations have shown that miR-92a acts as a cancer-causing agent or cancer suppressor in multiple human cancers, including non-small-cell lung cancer [24], colorectal cancer [34], and cervical cancer [33]. Nevertheless, the regulatory mechanism of miR-92a in the oncogenesis of OS is still not clear.

Dickkopf-related protein 3 (DKK3), an important member of the Dickkopf family, interacts with the Wnt signaling pathway and participates in a variety of biological processes, including embryogenesis and tumorigenesis [23,6,30]. Indeed, it has been reported that overexpression of DKK3 can effectively reduce the motility and invasion of OS cells [9]. Moreover, several studies have shown that DKK3 is a target of miR-92a [8,17]. This indicated that it would be meaningful to explore the functional roles of miR-92a and DKK3 in OS tumor progression.

In the present study, the expression of miR-92a in OS cells was analyzed, and its effects on the proliferation, apoptosis, migration, and invasion of OS cells were investigated. Furthermore, we confirmed that DKK3 was an immediate target of miR-92a and participated in the effects of miR-92a on the biological capacity of OS cells. Our results suggested that miR-92a could be a feasible target for clinical therapy of OS.

## Materials and methods

### Cell culture and transfection

 The normal human osteoblast line hFOB 1.19 and the OS cell lines U2OS, MG-63, MNNG, and 143B were acquired from the Shanghai Institute of Cell Biology, Chinese Academy of Sciences. The hFOB 1.19 cells and the four OS cell lines were cultured in high glucose DMEM supplemented with 10% fetal bovine serum, 100 U/ml streptomycin and 100 U/ml penicillin, and incubated at 37°C under a 5% CO_2_ water-saturated atmosphere.

MiR-92a mimics, miRNA negative control (miR-NC), miR-92a inhibitor, inhibitor negative control (NC), and siRNA for DKK3 (siDKK3) were acquired from GenePharma. Cells in the logarithmic growth stage were digested, counted, and inoculated into a six-well plate, ensuring 40–60% cell aggregation on the next day for transfection. Lipofectamine 2000 was used to perform cell transfection, and was mixed with the transfection reagents in serum-free medium in accordance with the manufacturer’s instructions. Quantitative real-time PCR (qRT-PCR) was used to verify the transfection efficiency.

### Total RNA extraction, reverse transcription, and qRT-PCR

Total RNA was extracted from cells using TRIzol reagent and reverse transcribed into cDNA using a PrimeScript first-strand cDNA synthesis kit, according to the manufacturer’s recommendation. Then, an ABI 7500 real-time PCR system was employed to carry out qRT-PCR using SyBR premix Ex Taq. U6 was used as an internal reference for miR-92a, while DKK3 was standardized to GAPDH. As shown in Table 1, Sangon Biotech (Shanghai, China) was commissioned to design and synthesize appropriate primers. The relative expression levels of miR-92a and DKK3 were calculated using the 2^−△△Ct^ method. All data were taken as the mean of three measurements.

**Table 1:** The qRT-PCR primer sequences

Gene	Primer sequences (5′–3′)
miR-92a	F: GCTGAGTATTGCACTTGTCCCG
	R: GTGTCGTGGAGTCGGCAA
U6	F: CTCGCTTCGGCAGCACA
	R: AACGCTTCACGAATTTGCGT
DKK3	F: AGGACACGCAGCACAAATTG
	R: CCAGTCTGGTTGTTGGTTATCTT
GAPDH	F: GGAGCGAGATCCCTCCAAAAT
	R: GGCTGTTGTCATACTTCTCATGG

### Protein extraction and Western blotting

 Protein was extracted from cells using RIPA lysis buffer and was quantified with a bicinchoninic acid assay kit. Then, protein (30 μg) was resolved by 10% SDS–PAGE and transferred onto polyvinylidene difluoride (PVDF) membranes followed by blocking with phosphate buffer saline with 0.1% Tween-20 (PBST) containing 5% non-fat dried milk, for 3 h, and incubating with primary antibodies (anti-DKK3, 1:1,000; anti-GAPDH, 1:1,000) at 4°C overnight. Membranes were then rinsed with TBST three times and subsequently incubated with secondary goat anti-rabbit IgG (1:2000) at 37°C for 30 min, then visualized using an ECL kit. Image J software was used to present grayscale and optical density.

### Cell proliferation assays

The cell counting kit-8 assay (CCK-8) and colony formation assay were used to evaluate cell proliferative capacity. Cells transfected for 24 h were digested and counted, then 2000 cells were inoculated into each well of a 96-well culture plate. The CCK-8 assay was performed every 24 h for the first 5 days after inoculation. The CCK-8 solution (10 μl) was added to each well at 24 h and allowed to react for 3 h. Absorbance was measured at 450 nm using a Microplate autoreader.

At 24 h after transfection, the trypsinized cells were collected and 1000 cells were inoculated into each well of a 12-well culture plate for colony formation assay. After 7 days, the cells were fixed with 4% paraformaldehyde at room temperature, then stained with 0.5% crystal violet and photographed, and colonies were counted.

### Apoptosis detection by flow cytometry

OS cells were digested and collected after transfection with corresponding reagents and cultured for 48 h. Then, the apoptosis rate of cells was analyzed by flow cytometry using an Annexin V-FITC/PI double staining kit in accordance with the manufacturer’s suggestions.

### Cell migration assay and cell invasion assay

Transwell assays were performed to assess the cells’ capacity for migration and invasion, and were conducted using Transwell chambers (pore size: 8 μm) and Millicell chambers (pore size: 8 μm). An OS cell suspension (1.5 × 10^6^ cells/ml) was prepared in serum-free DMEM and 200 μl was inoculated into the upper chamber, while 500 μl complete medium was inoculated into the lower chamber. After continuous culture for 24 h, the chambers were removed and the non-migratory or uninvaded cells were gently wiped off with a cotton swab, after which filters were fixed in 4% paraformaldehyde for 15 min followed by drying and staining with 0.5% crystal violet for 15 min at room temperature. An inverted microscope was used to photograph and count cells that had migrated or invaded.

### Dual-luciferase assay

The 293T cells in logarithmic growth phase were inoculated into a 24-well plate. Cells were subsequently co-transfected with miR-92a mimics, miR-NC, miR-92a inhibitor or inhibitor NC, and the plasmids containing wild-type (WT) or mutant (MUT) DKK3 3′-UTR, using Lipofectamine 2000. After transfection for 48 h, cells were collected and the Dual-Glo Luciferase Assay system was used to measure the luciferase activity in accordance with the manufacturer’s instructions, while the relative luciferase activity was presented as the ratio of Renilla luciferase activity to firefly luciferase activity (Table 2).

**Table 2:** Materials and manufacturers

Materials	Manufacturers
High glucose DMEM	Hyclone, Logan, UT, U.S.A.
Fetal bovine serum	Gibco/Life Technologies, Carlsbad, CA, U.S.A.
SiRNA	GenePharma, Shanghai, China
MiR-92a-antagomir, antagomir NC	GenePharma, Shanghai, China
Lipofectamine 2000	Invitrogen, Thermo Fisher Scientific, Waltham, MA, U.S.A.
TRIzol reagent	Thermo Fisher Scientific, Inc.
PrimeScript first-Strand cDNA Synthesis kit	Takara Bio, Shiga, Japan
SyBR premix Ex Taq	Takara Bio, Shiga, Japan
ABI 7500 real-time PCR system	Applied Biosystems. Foster City, CA, U.S.A.
RIPA lysis buffer	Beyotime Institute of Biotechnology, Jiangsu, China
Bicinchoninic acid assay kit	Beyotime Institute of Biotechnology, Jiangsu, China
CCK-8 assay kit	Beyotime Institute of Biotechnology, Jiangsu, China
Anti-DKK3	Abcam (ab186409), Cambridge, MA, U.S.A.
Anti-GAPDH	Cell Signaling Technology (D16H11), Inc, China
ECL kit	Thermo Fisher Scientific, Inc.
Image J software	NIH, Bethesda, MD, U.S.A.
Microplate Autoreader	Bio-Rad, Hercules, CA, U.S.A.
Annexin V-FITC/PI double staining kit	BD Biosciences, Franklin Lakes, NJ, U.S.A.
Transwell chambers	Costar, Corning Inc., Corning, NY, U.S.A.
Millicell chambers	Merck KGaA, Darmstadt, Germany
Dual-Glo Luciferase Assay system	Promega Corp., Madison, WI, U.S.A.

### *In vivo* experiment

In the experiment of subcutaneous tumor formation, we chose 5-week-old male athymic BALB/c nude mice (*n*=6) for research. An aliquot of 0.1 ml PBS containing 2 × 10^6^ MNNG cells was subcutaneously injected into the posterior flanks of mouse (each mouse receive dtwo injection)s. The tumor volume was measured with a caliper every week, using the standard formula: tumor volume (mm^3^) = length × width^2^/2. When the tumors reached a volume of approximately 100 mm^3^, the mice were treated with local injection of miR-92a-antagomir or miR-92a-antagomir NC (200 pmol per injection) into multiple sites of the xenograft tumors, injecting every 3 days. All mice were sacrificed 3 weeks later and tumor volume was recorded. The *in vivo* study was carried out in accordance with the agreement approved by the Animal Care Committee of Tongji University School of Medicine (SYXK 2014-0026).

### Statistical analysis

Western blotting, qRT-PCR, luciferase assay, and cell biological capability assays were performed in triplicate, repeating several times. Overall data are presented as mean ± SD. The significance of differences between the groups was evaluated using Student’s *t*test. Differences were considered statistically significant at *P*<0.05.

## Results

### MiR-92a is prominently overexpressed in OS cell lines

**Figure 1:**
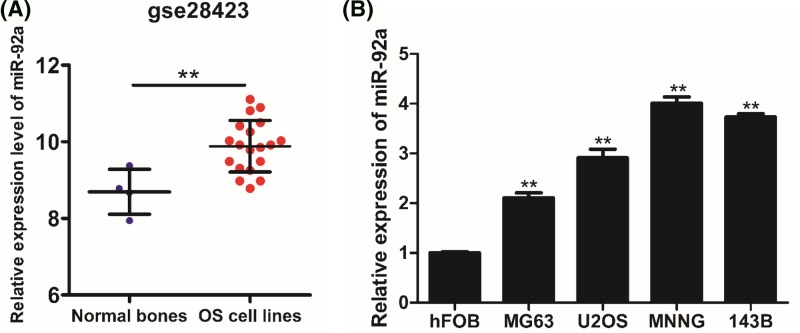
MiR-92a is prominently up-regulated in human OS cell lines (**A**) The expression level of miR-92a in GSE28423. (**B**) Relative expression level of miR-92a was assessed by qRT-PCR in OS cell lines (MG-63, U2OS, MNNG, and 143B) in comparison with hFOB1.19 cells. U6 was used as the standard reference. ^*^P<0.05 vs control; ^**^P<0.01 vs control.

At the beginning of the experiment, we analyzed the data in GSE28423 from the NCBI-Gene Expression Omnibus (GEO) database (https://www.ncbi.nlm.nih.gov/geo/), which contained 19 OS cell lines and four normal bones [21]. The expression of miR-92a was overexpressed in OS cell lines compared with normal bones (Figure 1A). Then, we further verified this analysis by qRT-PCR. As demonstrated in Figure 1B, miR-92a’s expression was remarkably increased in all four OS cell lines compared with normal human osteoblast cells.

### MiR-92a accelerates cell proliferation and impedes the apoptosis of OS cells

**Figure 2:**
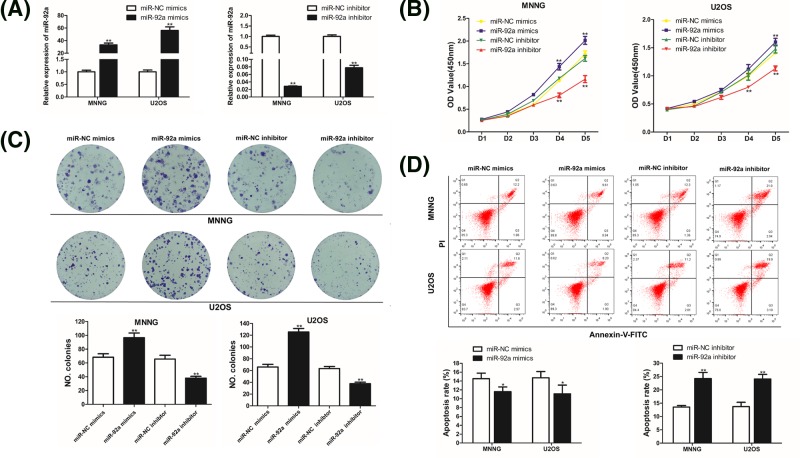
MiR-92a facilitates cell proliferation while suppressing apoptosis of OS cells *in vitro* MNNG and U2OS cells were transfected with miR-92a mimics, miR-92a inhibitor, or corresponding NC. (**A**) The miR-92a levels in MNNG and U2OS cells were detected by qRT-PCR after 48 h of transfection. (**B**) The CCK-8 assay was used to assess cell viability with absorbance measured at 450 nm. (**C**) Representative photographs and quantitative analyses of plate colony formation of MNNG and U2OS cells. (**D**) At 48 h after transfection, apoptosis was measured by flow cytometry. All assays were performed at least three times. Data are presented as the mean values ± SD. ^*^P<0.05 vs control; ^**^P<0.01 vs control.

In order to research the function of miR-92a in the development of OS, overexpression and inhibition experiments were performed. As shown in Figure 1B, MNNG and U2OS cells with higher expression levels of miR-92a were selected and separately transfected with miR-92a mimics, miR-92a inhibitor or corresponding NCs. After transfection for 48 h, qRT-PCR analysis showed that the transcription levels of miR-92a in both MNNG and U2OS cells were remarkably increased in the mimics group, while those in the inhibitor group were obviously decreased (vs control, Figure 2A).

CCK-8 and colony formation assays were subsequently applied to test the impacts of miR-92a on cell proliferation of OS. As shown in Figure 2B and C, cell growth in the group transfected with miR-92a mimics was remarkably increased in comparison to the NC group. In contrast, the growth of MNNG and U2OS cells was prominently inhibited in the miR-92a inhibitor transfected group compared with the NC group. These data revealed that up-regulation of miR-92a promoted proliferation of OS cells, while its down-regulation suppressed OS cell proliferation.

Considering that miR-92a regulates cell proliferation, we hypothesized that miR-92a is also involved in the apoptosis process of OS cells. Consequently, we performed flow cytometric analysis to clarify the impact of miR-92a on OS cell apoptosis. As demonstrated in Figure 2D, increased expression of miR-92a reduced the apoptosis rate of MNNG and U2OS cells in comparison with the NC group, while suppression of miR-92a obviously increased apoptosis of MNNG and U2OS cells. These results revealed the characteristic of miR-92a in impeding apoptosis of OS cells.

### MiR-92a facilitates the migration and invasion of OS cells

**Figure 3:**
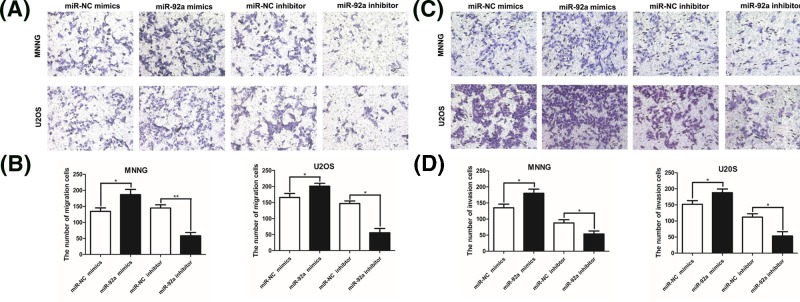
MiR-92a promotes the migration and invasion of OS cells MNNG and U2OS cells were transfected with miR-92a mimics, miR-92a inhibitor, or corresponding NC. (**A, B**) Transwell assays were used to evaluate cell migration after 48 h of transfection. Cells that traveled through the microporous membrane are shown in photographs (A) and the numbers of migrated cells are shown in a histogram (B). (**C, D**) Invasion ability was also detected after transfection for 48 h using a Transwell assay. Values represent the mean ± SD (*n*=3 replicates). ^*^P<0.05 vs control; ^**^P<0.01 vs control. NC, normal control.

The influence of miR-92a on the migration and invasion of OS cells was investigated by Transwell assays. The results indicated that up-regulation of miR-92a prominently enhanced the migration ability of MNNG and U2OS cells, while silencing of miR-92a remarkably lessened the migration capacity of both cell lines (Figure 3A,B). Likewise, similar findings were observed in the invasion assay (Figure 3C,D). Based on these results, we concluded that miR-92a promoted the migration and invasion of OS cells.

### Inhibition of miR-92a suppresses the growth of OS cells *in vivo*

**Figure 4:**
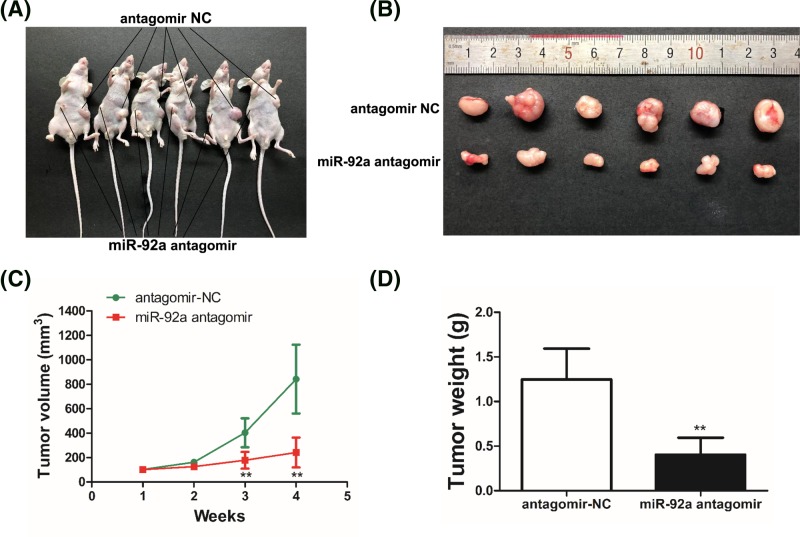
The growth of OS *in vivo* was suppressed by the inhibition of miR-92a (**A**) Photograph of nude mice, which were injected with MNNG cells into either side of the posterior flank subcutaneously and received different treatments with either miR-92a-antagomir or miR-92a-antagomir NC. (**B**) Photograph showing the tumors harvested from nude mice in the MNNG/miR-92a-antagomir group and in the MNNG/miR-92a-antagomir NC group. (**C**) The growth curve of subcutaneous xenografts of MNNG cells. Tumor diameters were measured every week. (**D**) Comparison of the average weight of tumors between the two groups. Data represent the mean ± SD (*n*=6); ^**^P<0.01 vs control.

Next, we investigated whether miR-92a could regulate the tumor growth of OS in mouse using the subcutaneous tumor formation assay. As expected, we observed that tumors grew slower and were remarkably smaller in the MNNG/miR-92a-antagomir group than those in the MNNG/miR-92a-antagomir NC group (Figure 4). Based on the above results, we further confirmed that miR-92a functions as an oncogene, which facilitates the tumor growth, while its inhibitor suppresses the tumorigenesis and development of OS in nude mice.

### MiR-92a directly targets DKK3 by negatively modulating the expression DKK3 in OS cells

**Figure 5:**
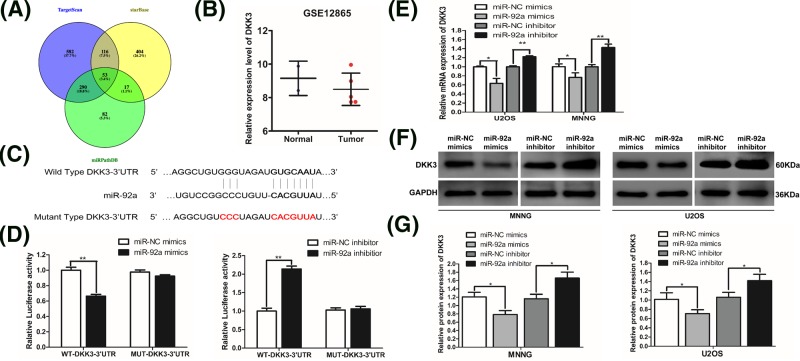
DKK3 is the target of miR-92a in human OS cells (**A**) Veen diagram for the targets of miR-92a in three databases. (**B**) The expression level of DKK3 in GSE12865. (**C**) The nucleotide binding sites in the 3′-UTR sequence of DKK3 for miR-92a. The construction of WT and MUT DKK3 luciferase plasmids. (**D**) A dual-luciferase reporter assay system was used to analyze the relative luciferase activity at 48 h after transfection. (**E**) The mRNA levels of DKK3 in MNNG and U2OS cells after transfection with corresponding reagents. (**F**) Western blotting for DKK3 in MNNG and U2OS cells at 48 h after transfection. (**G**) Relative protein expression of DKK3 in MNNG and U2OS. All data are representative of the mean of three independent assays and are presented as the mean ± SD. ^*^P<0.05 vs control; ^**^P<0.01 vs control.

To further explore the mechanism of miR-92a in OS, analysis by bioinformatics methods (TargetScan, starBase, and miRPathDB) was performed to verify the downstream targets of miR-92a. Thousands of downstream targets were predicted. By overlapping three prediction lists, we found that 53 targets were coincident (Figure 5A, Supplementary Table S1). Then, we continued to narrow down the range of candidate targets through consulting the literature, and focused on targets that were mostly involved in cell proliferation, apoptosis, migration, and invasion. Interestingly, Hoang *et al.* [9] found that DKK3 had an inhibitory effect on invasion and motility of OS cells. But, the relationship between miR-92a and DKK3 has not been studied. The analysis of data in GSE12865 from GEO database indicated that the expression of DKK3 in OS tissues was lower than that in normal bones (Figure 5B) [27]. Therefore, we further investigated whether miR-92a regulated OS cells through DKK3.

As shown in Figure 5C, DKK3 is a feasible target of miR-92a, with ten nucleotide sites in the 3′-UTR region of DKK3 mRNA that can bind to miR-92a. Analyses by luciferase assays revealed that the relative luciferase activity in 293T cells co-transfected with miR-92a mimics and WT DKK3 3′-UTR luciferase plasmid was significantly lower than that in the NC group. (Figure 5D). However, no significant difference in luciferase activity was observed in the group co-transfected with miR-92a mimics and MUT DKK3 3′-UTR plasmid compared with the NC group (Figure 5D). In contrast, we found that the relative luciferase activity in 293T cells co-transfected with miR-92a inhibitor and WT DKK3 3′-UTR luciferase plasmid was remarkably increased in contrast with the NC group, while there was no significant difference between the two groups transfected with MUT DKK3′-UTR luciferase plasmid.

In addition, we analyzed the correlation between miR-92a and DKK3 in MNNG and U2OS cells. The qRT-PCR and Western blotting analyses revealed that the expression of miR-92a was negatively correlated with the expression of DKK3. As shown in Figure 5E,G, the mRNA and protein levels of DKK3 were both reduced by overexpression of miR-92a, while knockdown of miR-92a significantly enhanced both the mRNA and protein levels of DKK3 in comparison with the NC group. In summary, these results suggested that miR-92a down-regulates the expression of DKK3 by directly binding to its 3′-UTR sequence.

### DKK3 participates in the regulation of miR-92a on the biological function of OS cells

**Figure 6:**
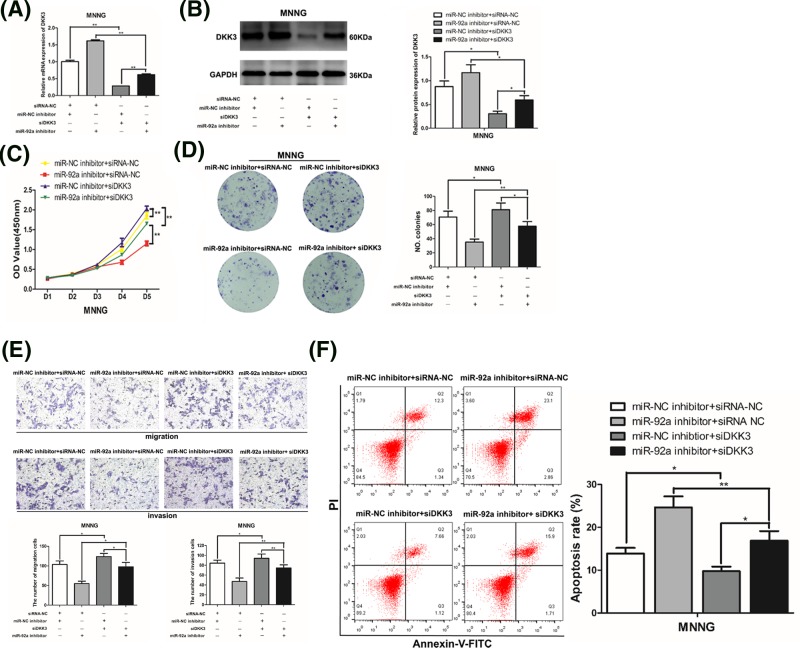
Down-regulation of DKK3 partly rescues the inhibitory effects on OS cells induced by miR-92a inhibitor MNNG cells were transfected with miR-92a inhibitor or miRNA-NC inhibitor and siDKK3 or siRNA-NC. (**A**) The mRNA level of DKK3 in MNNG cells was evaluated by qRT-PCR after 48 h of transfection. (**B**) The results of Western blotting for DKK3 in MNNG cells. The proliferation of MNNG cells was identified by CCK-8 assay (**C**) and colony forming assay (**D**). (**E**) The migration and invasion of MNNG cells was analyzed by Transwell assays. (**F**) Apoptosis of MNNG cells was analyzed by flow cytometry. Data from three independent experiments are expressed as the mean ± SD; *P<0.05 vs control; **P<0.01 vs control.

Because DKK3 is the target gene of miR-92a in OS cells, we further explored whether DKK3 participates in the carcinogenic effects of miR-92a on OS cells. We used MNNG cells to transfect with siDKK3 (siDKK3-1 and siDKK3-2) or siRNA-NC, and selected siDKK3-1 for its better transfection efficiency (Supplementary Figure S1). Then, MNNG cells were co-transfected with miR-92a inhibitor or miR-NC inhibitor and siDKK3 (siDKK3-1) or siRNA-NC. As shown in Figure 6A,B, the mRNA and protein levels of DKK3 in the miR-NC inhibitor + siDKK3 group were lower than those in the miR-NC + siRNA-NC group, indicating the successful transfection of siDKK3. The expression of DKK3 in the miR 92a inhibitor + siDKK3 group was higher than that in the miR-NC inhibitor + siDKK3 group, but less than that of the miR-92a inhibitor + siRNA NC group.

In terms of cell function experiments, the results revealed that knockdown of DKK3 promoted cell proliferation (Figure 6C,D) and metastasis (Figure 6E,F), while reducing the apoptosis rate of MNNG cells (Figure 6G). Of importance, the inhibition of miR-92a increased expression of DKK3 and weakened the stimulatory effect on tumorigenesis induced by siDKK3. Furthermore, the results also demonstrated that silencing of DKK3 partly reversed the suppressive effects on cell proliferation (Figure 6C,D) and metastasis (Figure 6E,F) induced by miR-92a inhibitor, and also partially impeded the apoptosis of MNNG cells promoted by miR-92a inhibitor (Figure 6G). The above data implied that miR-92a plays a carcinogenic role, partially by suppressing DKK3 in OS cells. Additionally, miR-92a inhibitor effectively restrained the cell biological behavior of OS and promoted apoptosis of OS cells, through its transcriptional modulation of DKK3.

## Discussion

MiRNAs play a crucial role in cellular biological processes, and their aberrant expression is usually linked with the occurrence and development of tumors, such that they function as carcinogenes or tumor suppressor genes [5,4]. Consequently, the identification of specific miRNAs and their targets could provide important clues for the diagnosis and treatment of cancers. Among the plentiful miRNAs known, miR-92a has been proven to be a significant oncogene and prognostic marker of human malignant tumors. For example, miR-92a is known to be overexpressed in colorectal cancer [34]. Ren *et al.* [24] discovered that miR-92a promoted growth, metastasis, and chemoresistance in non-small-cell lung cancer cells by targeting PTEN. Moreover, Yu and colleagues [33] reported that overexpression of miR-92a promoted the proliferation and invasion of cervical cancer cells by targeting FBXW7. This investigation extended the role of miR-92a in cancers, suggesting that miR-92a plays a role as a carcinogenic miRNA in OS.

In our investigation, miR-92a was significantly up-regulated in OS cell lines in comparison with a normal osteoblastic line, which was in line with former research reported by Gougelet *et al.*, Namløs *et al.*, and *Li et al.* [7,21,15]. We also confirmed that miR-92a promoted the proliferation and metastasis, and suppressed the apoptosis of OS cells. In addition, miR-92a inhibitor exerted an inhibitory effect on the growth of OS cells both *in vitro* and *in vivo.*

Since the authentication of target genes is the pivotal step in evaluating the function of miRNAs that are abnormally expressed in cancers, we applied three bioinformatics programs to predict the latent targets of miR-92a, finally focused on DKK3. DKK3, also known as Reduced Expression in Immortalized Cells (REIC), has been deemed to function as an important cancer suppressor which down-regulated in quite a few cancers [12,29,20]. Two studies reported that down-regulation of DKK3 was bound up with poor clinical prognosis of cervical cancer [26], while up-regulation of DKK3 inhibited the proliferation of cervical cancer cells [13]. Interestingly, Hoang *et al.* [9] indicated that DKK3 suppressed the invasion and motility of Saos-2 OS cells by regulating the Wntβ–catenin pathway. In the present study, we found that DKK3 had nucleotide binding sites in its 3′-UTR for miR-92a. Our luciferase assay confirmed that miR-92a led to a significant reduction of luciferase activity in the WT DKK3 3′-UTR reporter, but had no inhibitory effect on those in the MUT DKK3 3′-UTR reporter. In addition, we verified that expression of miR-92a negatively modulated the expression level of DKK3. Silencing of miR-92a resulted in increased expression of DKK3 at both protein and mRNA levels in MNNG and U2OS cells, while up-regulation of miR-92a remarkably lowered the protein and mRNA levels of DKK3. These data validated reports that miR-92a restrains the transcription expression of DKK3 by targeting its 3′-UTR region directly. Interestingly, the targeted relationship between miR-92a and DKK3 has also been demonstrated in cervical cancer and neuroblastoma [8,17]. Thus, our data build on previous studies on the regulation of DKK3 by miR-92a in human cancers.

To further research the interaction between mir-92a and DKK3, siDKK3 was applied in MNNG cells to rescue the functional phenotype produced by mir-92a inhibitor. Silencing of DKK3 partly alleviated the inhibition of tumor growth induced by miR-92a inhibitor. Additionally, suppression of miR-92a promoted the expression of DKK3 and reduced the auxo-action of siDKK3 on the growth of MNNG cells. Therefore, our results revealed that miR-92a inhibitor could restrain the malignancy of OS at least partially by up-regulating DKK3.

## Conclusion

Taken together, our findings confirmed that miR-92a facilitated the biological behavior of OS cells and reduced apoptosis by suppressing DKK3. Moreover, the miR-92a inhibitor impeded the tumorigenesis of OS by activating DKK3. This will help improve our understanding of the etiopathogenesis of OS and offer a novel therapeutic target and evaluation marker for OS.

## Supporting information

**Supplementary Figure 1:** The comparison of two siRNAs for DKK3 (siDKK3-1, siDKK3-2) was detected by qRT PCR. The efficiency of siDKK3-1 is better than siDKK3-2.

**Supplemental Table S1:** 53 coincident targets obtained by overlapping three predicted lists.

## Abbreviations

CCK: cell counting kit
DKK3: Dickkopf-related protein 3
GAPDH: Glyceraldehyde-3-phosphate dehydrogenase
GEO: Gene Expression Omnibus
miR-NC: miRNA negative control
MUT: mutant
NC: negative control
OS: osteosarcoma
qRT-PCR: quantitative real-time PCR
RIPA: Radio Immunoprecipitation Assay
siDKK3: siRNA for DKK3
WT: wild-type
