# Supplementary material for: MiR-92a modulates proliferation, apoptosis, migration, and invasion of osteosarcoma cell lines by targeting Dickkopf-related protein 3
Source: Biosci Rep. 2019 Apr 26;39(4):BSR20190410. doi: 10.1042/BSR20190410 (PMC6487267; doi:10.1042/BSR20190410)
Supplement: Supplementary file 1 [file bsr20190410_Supp1.pdf]

Figure S1. The comparison of two siRNAs for DKK3 (siDKK3-1, siDKK3-2) was detected by qRT-PCR. The efficiency of siDKK3-1 is better than siDKK3-2.

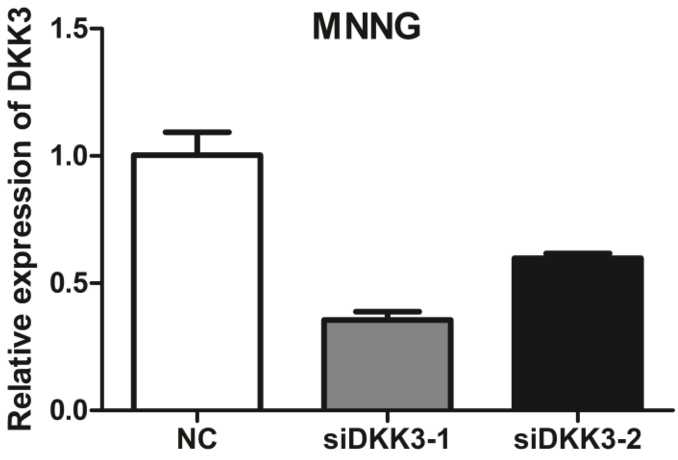

Table S1. 53 coincident targets obtained by overlapping three predicted lists.

**53 overlapping targets**							
CD69	DCAF6	HIPK3	BTG2	PPCS	ERGIC2	ANP32E	DUSP10
DKK3	ADRB1	DUSP5	RSBN1	RNF141	PLEKHA1	MIA3	CPEB3
MCOLN2	LMBR1L	AIDA	PIK3R3	ITGA5	CDKN1C	FAR1	OTUD3
BSDC1	ZDHHC5	RGL1	ZFC3H1	SESN3	PTEN	KLF6	LMO2
DENND4B	FNBP4	ARHGAP29	TWF1	PIK3AP1	GRAMD1B	FMN2	UBASH3B
KIF5B	SLC25A16	HIPK1	QSER1	SLC38A2	ATP2B4	FAM160B1	S1PR1
LUZP1	MAPK8	ANK3	CCSER2	PGAM1			
